# Hepatitis B Surface Antigen Seroprevalence among Prevaccine and Vaccine Era Children in Bangladesh

**DOI:** 10.4269/ajtmh.17-0721

**Published:** 2018-07-16

**Authors:** Repon C. Paul, Mahmudur Rahman, Eric Wiesen, Minal Patel, Kajal C. Banik, Ahmad R. Sharif, Sharmin Sultana, Mizanur Rahman, Jayantha Liyanage, Nihal Abeysinghe, Saleem Kamili, Trudy Murphy, Stephen P. Luby, Eric E. Mast

**Affiliations:** 1 icddr,b, Dhaka, Bangladesh;; 2 School of Public Health and Community Medicine, University of New South Wales, Sydney, Australia;; 3 Institute of Epidemiology, Disease Control and Research, Dhaka, Bangladesh;; 4 Centers for Disease Control and Prevention, Atlanta, Georgia;; 5 World Health Organization, South-East Asia Regional Office, New Delhi, India;; 6 Stanford University, Stanford, California

## Abstract

Bangladesh introduced hepatitis B vaccine in a phased manner during 2003–2005 into the routine childhood vaccination schedule. This study was designed to evaluate the impact of the introduction of hepatitis B vaccine in Bangladesh by comparing hepatitis B surface antigen (HBsAg) prevalence among children born before and after vaccine introduction and to estimate the risk of vertical transmission of chronic hepatitis B virus (HBV) infection from mother to infant. We also evaluated the field sensitivity and specificity of an HBsAg point-of-care test strip. We selected a nationally representative sample of 2,100 prevaccine era and 2,100 vaccine era children. We collected a 5-mL blood sample from each child. One drop of blood was used to perform rapid HBsAg testing. If a child had a positive HBsAg test result with the rapid test, a blood sample was collected from the mother of the HBsAg-positive child and from the mothers of two subsequently enrolled HBsAg-negative children. All samples were tested for serologic markers of HBV infection using standard enzyme-linked immunosorbent assay. One (0.05%) child in the vaccine era group and 27 (1.2%; 95% confidence interval [CI]: 0.8–1.7%) children in the prevaccine era group were HBsAg positive. Mothers of HBsAg-positive children were more likely to be HBsAg positive than mothers of HBsAg-negative children (odds ratios = 4.7; 95% CI: 1.0–21.7%). Sensitivity of the HBsAg rapid test was 91.2% (95% CI: 76.6–98.1%) and specificity was 100% (95% CI: 99.9–100%). The study results suggest that even without a birth dose, the hepatitis B vaccine program in Bangladesh was highly effective in preventing chronic HBV infection among children.

## INTRODUCTION

Hepatitis B virus (HBV) infection is a leading cause of morbidity and mortality worldwide because of hepatocellular carcinoma and liver cirrhosis.^1^ Globally, about 248 million people are chronically infected with HBV^2^; an estimated 686,000 HBV-related deaths occur, most of which result from the complications of chronic HBV infection, for example, liver failure and hepatocellular carcinoma.^3^ Hepatitis B virus is transmitted by percutaneous and permucosal exposure to infected blood and other body fluids.^4^ In highly endemic countries (hepatitis B surface antigen [HBsAg] prevalence ≥ 8%), the most common routes of transmission of HBV are from mother to child at birth or from person to person in early childhood.^5–7^ In low endemic countries, mother-to-child transmission or early childhood transmission may also account for more than one-third of chronic HBV infections.^8,9^

Preventing HBV infections acquired at birth and in early childhood is critical, as the risk of becoming chronically infected is 90% if infected at birth or up to 6 months of age, 20–60% if infected between 6 months and 5 years of age, and 5–10% if infected after 5 years of age.^10–12^ As recommended by the World Health Organization (WHO) in 1992, nearly all countries globally have introduced hepatitis B vaccine into national infant immunization schedules to prevent chronic HBV infection and the associated disease burden.^13^ Inclusion of hepatitis B vaccine in infant immunization schedules has led to a marked reduction in the prevalence of chronic HBV infection and in the rate of hepatocellular carcinoma in countries where chronic HBV infection is highly endemic.^14,15^ Since 2009, WHO recommends administering the first dose of hepatitis B vaccine as soon as possible after birth (within 24 hours) for optimal prevention of mother-to-child transmission and control of HBV transmission, regardless of the level of HBsAg endemicity.^4^

Hepatitis B vaccine was introduced in Bangladesh in a phased manner during 2003–2005 into the Expanded Program on Immunization, using the WHO-recommended schedule at 6, 10, and 14 weeks of age. The hepatitis B vaccine birth dose was not introduced into the childhood vaccination schedule; 71% of births in Bangladesh occurred at home in 2011, which is a logistic barrier to administering a timely birth dose of hepatitis B vaccine.^16^ Although data on the burden of chronic HBV infection in Bangladesh are limited, previous small-scale studies evaluated the prevalence of HBsAg, a marker of chronic HBV infection, to be 3–7% among the general population^17–20^ and 1.5–12% among children aged < 5 years.^17,18,20^

This study was designed to evaluate the impact of the introduction of hepatitis B vaccine in Bangladesh by comparing HBsAg prevalence among children born before and after hepatitis B vaccine introduction and to estimate the risk of vertical transmission of chronic HBV infection from mother to child in Bangladesh. We also evaluated the field sensitivity and specificity of an HBsAg rapid point-of-care test strip.

## METHODS

### Serosurvey.

**Figure 1.**
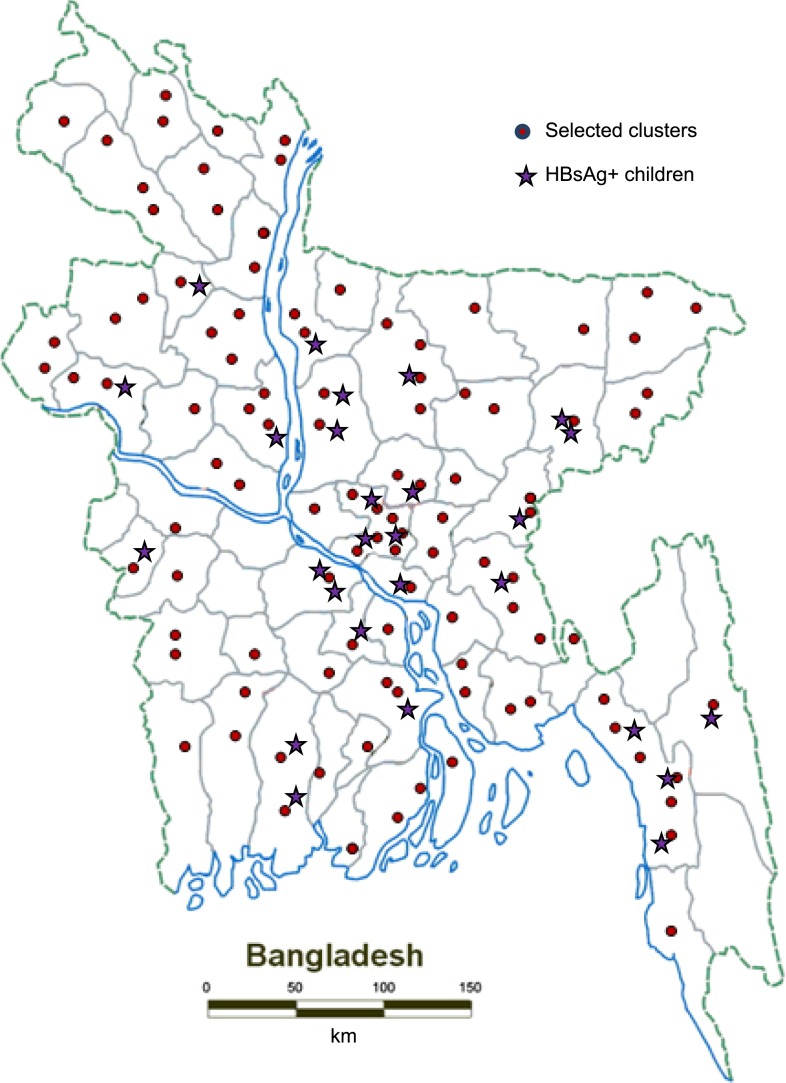
Clusters selected and location of hepatitis B surface antigen–positive (HBsAg+) children, hepatitis B seroprevalence study—Bangladesh, 2011–2012. This figure appears in color at www.ajtmh.org.

We selected a nationally representative sample of 2,100 children in two age groups. The first group included children born from April 1, 2001 to March 31, 2002 (before the introduction of hepatitis B vaccine). The second age group included children born from November 1, 2005 to October 31, 2006 (immediately after the introduction of the vaccine). The sample size was calculated by assuming HBsAg prevalence for the prevaccine era children to be 3.5% and for the vaccine era children to be 1.5%, and by considering 80% power, 5% precision, and a design effect of two to take into account possible homogeneity due to cluster sampling. A two-stage cluster design was used. In the first stage, 105 *mouzas* (lowest enumeration area in both rural and urban areas) were randomly selected from a complete list of > 62,000 *mouzas* in Bangladesh using the probability proportional to size (Figure 1). According to the 2001 population census, each *mouza* has an average population of about 2,000.^21^ At the second stage, 20 children per age group were selected from each *mouza* using a modified random walk approach.

In each *mouza*, two field teams, each comprising one interviewer and one phlebotomist, worked simultaneously to search for eligible children. One team searched from the center of the *mouza* to the peripheral boundary in a randomly chosen direction and the other team searched from the opposite direction. Households were visited until the teams found 20 prevaccine era and 20 vaccine era children. If the required number of eligible children was unavailable in the selected *mouza*, the team visited the adjacent *mouza* to enroll the remaining children. Only one child was selected from a household. The age of eligible children was verified by reviewing the child vaccination card or birth registration certificate, if available. If neither vaccination card nor birth certificate was available, the team verified the age of children by consulting with their guardians. Interviewers collected information on children’s demography, vaccination history, and possible risk exposures for HBV infection. Vaccination history was obtained from children’s vaccination cards or by their guardians’ reports if vaccination cards were unavailable.

### Sample collection and HBsAg point-of-care testing.

In each *mouza*, blood sample collection sites were set up temporarily in community health clinics or houses of local leaders. Guardians of selected children were requested to bring their children to sample collection sites. If any guardians were reluctant to bring their children to sample collection sites, the team visited their households and collected blood samples from children. Phlebotomists collected a 5-mL venous blood sample from children after obtaining written consent from guardians and verbal consent from children.

Immediately after collection of blood samples from children, we performed point-of-care HBsAg testing, with one drop (∼50 μL) of collected blood sample using Determine^™^ HBsAg point-of-care test strips manufactured by Alere Medical Co. Ltd., Japan. The reported sensitivity and specificity of Determine^™^ HBsAg range from 95% to 100% and 96–100%, respectively.^22–24^ One drop of anticoagulant buffer was added to the drop of whole blood as required by the manufacturer (anticoagulant buffer is not required if the point-of-care testing is performed with serum in laboratory settings^22^). In the initial stage of the study, 270 rapid HBsAg tests were performed using serum from the blood sample until anticoagulant buffer was available. However, if any child was found HBsAg positive by the laboratory assay, the team went back to the child to perform the rapid test using whole blood.

If a child had a positive HBsAg test result with the rapid test, a 5-mL venous blood sample was collected from the mother of the HBsAg-positive child and from the mothers of two subsequently enrolled HBsAg-negative children after obtaining written consent. The use of HBsAg point-of-care testing enabled field teams to provide test results to participants immediately and to collect blood samples from mothers of HBsAg-positive children in a single visit. All participants were informed of the HBsAg test results. A brochure was supplied to participants with information on what to do if they were HBsAg positive.

### Sample processing and laboratory testing.

Each day, phlebotomists centrifuged blood specimens at the Upazila (subdistrict) Health Complex; two aliquots of extracted sera were prepared from each specimen and stored at 2–4°C in the refrigerator of the Upazila Health Complex. All samples were transported at 2–4°C to the laboratory of the Institute of Epidemiology, Disease Control and Research (IEDCR) in Dhaka within 1 week of collection. We monitored the temperature of collected samples beginning from the time of collection through transport to IEDCR laboratory using a temperature data logging device (LogTag Recorders Limited, Auckland, New Zealand).

**Figure 2.**
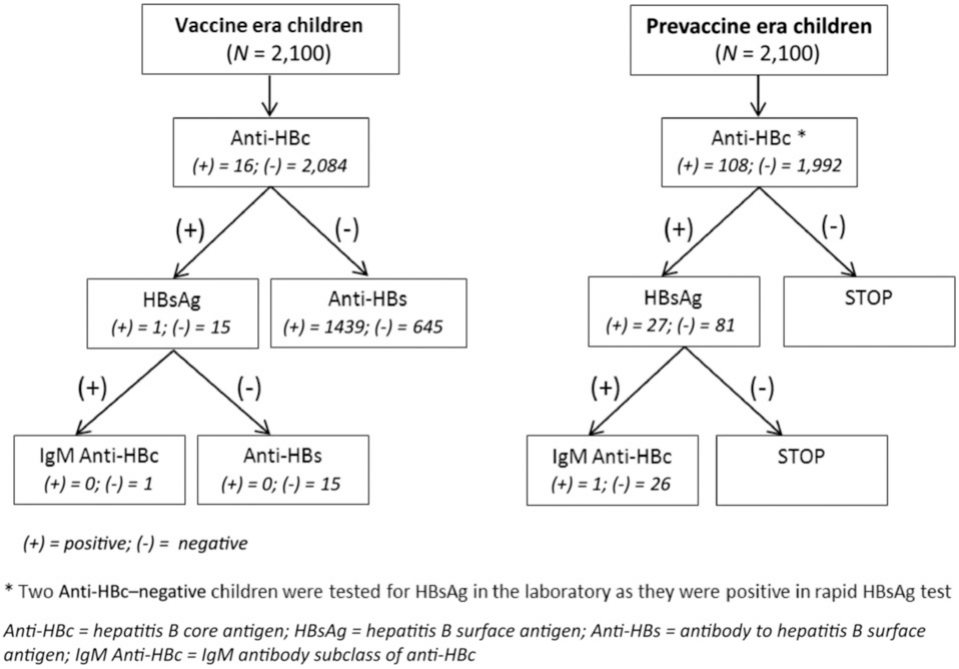
Algorithm for testing children’s serum samples for markers of infection, hepatitis B seroprevalence study—Bangladesh, 2011–2012.

**Figure 3.**
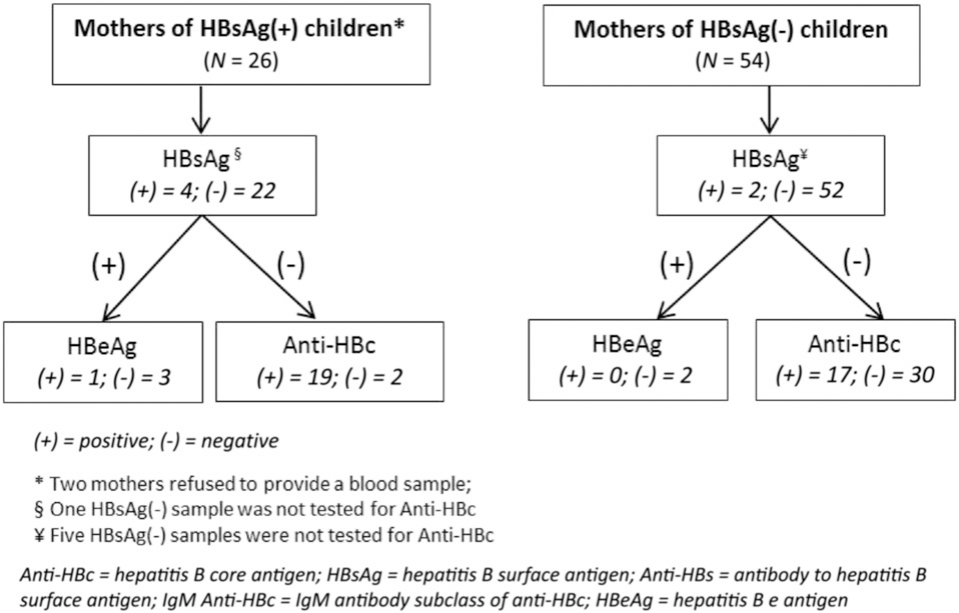
Algorithm for testing mothers’ serum samples for markers of infection, hepatitis B seroprevalence study—Bangladesh, 2011–2012.

Samples were tested at the IEDCR laboratory for HBsAg using enzyme-linked immunosorbent assay (ELISA) kits manufactured by Biomerieux, France; antibody to HBsAg (anti-HBs) (a marker of response to vaccination or recovery from HBV infection) using ELISA kits manufactured by Biokit, Spain; antibody to hepatitis B core antigen (anti-HBc) (a marker of recent or past HBV infection), immunoglobulin M (IgM) anti-HBc (a marker of recent or acute HBV infection), and hepatitis B e antigen (HBeAg) (a marker of high-level viremia) using ELISA kits manufactured by DiaSorin, Italy. All samples from children were first tested for anti-HBc and all anti-HBc–positive samples were then tested for HBsAg. In addition, all positive samples by HBsAg point-of-care testing, irrespective of anti-HBc test result, were tested for HBsAg by ELISA. The samples were tested for anti-HBs and IgM anti-HBc according to a laboratory algorithm (Figure 2). All samples from mothers were tested first for HBsAg; HBsAg-positive samples were then tested for HBeAg and HBsAg-negative samples were tested for anti-HBc (Figure 3).

As part of quality assurance of laboratory testing, all HBsAg-positive samples and 20% of HBsAg-negative samples (selected randomly) were also tested for HBsAg at the Division of Viral Hepatitis Laboratory of the U.S. Centers for Disease Control and Prevention (CDC), Atlanta, GA. The result obtained from the CDC laboratory was considered final if HBsAg results from the two laboratories were discordant. The field team informed the guardian of children of any corrected HBsAg results.

### Data analysis.

Hepatitis B virus infection was defined as a positive HBsAg result or a positive anti-HBc result. Chronic HBV infection was defined as a positive HBsAg result and a negative IgM anti-HBc result. Vaccine-induced immunity was defined as a positive anti-HBs result and a negative anti-HBc result.

We calculated the prevalence of HBsAg and anti-HBc by prevaccine and vaccine era group. Percentages with a 95% confidence interval (CI) were used to describe the prevalence. Bivariate analysis was performed to identify the potential risk factors for HBV infection among the prevaccine era children. Odds ratios (ORs) with a 95% CI were used to describe associations. We used cluster-robust variance estimation to account for cluster effect in estimating 95% CIs of HBsAg and anti-HBc prevalence, and ORs of risk factors, assuming the sample was self-weighting.

We used a conditional logit model to estimate the contribution of vertical transmission from mother to infant to the burden of chronic HBV infection by comparing the prevalence of HBsAg among mothers of HBsAg-positive children and among mothers of HBsAg-negative children. We calculated the sensitivity and specificity of HBsAg point-of-care testing using ELISA test results as the gold standard. Confidence interval of sensitivity and specificity of HBsAg point-of-care testing was calculated using exact binomial method. The analysis was carried out using ©STATA version 13 (StataCorp., College Station, TX).

### Human subjects.

The field teams obtained verbal consent from the participating children and written consent from the guardians. The study protocol was reviewed and approved by the institutional review boards of the icddr,b and the U.S. CDC.

## RESULTS

### Survey implementation.

The field work took place from September 2011 to April 2012. Of the 64 total districts in Bangladesh, 58 were represented in the survey (Figure 1). The field teams approached 2,203 prevaccine era children and 2,270 vaccine era children to enroll 2,100 children from each group; 103 (5%) of the prevaccine era children and 170 (8%) of the vaccine era children or their guardians refused to provide blood samples. One cluster was replaced because it was inaccessible because of security issues.

### Background characteristics and vaccination status of children.

The median age of enrolled prevaccine era children was 10.2 years (interquartile range [IQR] = 10.0–10.4) and the median age of vaccine era children was 5.7 years (IQR = 5.4–5.9). Both groups had similar proportions of children by gender, residence, religion, and household expenditure characteristics. However, mothers of prevaccine era children had less education than the mothers of vaccine era children (*P* < 0.01) (Table 1).

**Table 1:** Background characteristics and vaccination status of participating children, hepatitis B seroprevalence study—Bangladesh, 2011–2012

Characteristics	Prevaccine era children* (*N* = 2,100) *n* (%)	Vaccine era children† (*N* = 2,100) *n* (%)
Background characteristics of children		
Gender, boys	1,085 (51.7)	1,077 (51.3)
Residence		
Rural	1,680 (80.0)	1,680 (80.0)
Urban	420 (20.0)	420 (20.0)
Median household expenditure in Bangladeshi Taka (IQR)	6,000 (5,000–10,000)	6,000 (5,500–12,000)
Mothers’ education		
No education	660 (31.3)	480 (22.9)
Some primary (1–4 class)	511 (24.3)	447 (21.3)
Completed primary (5–9 class)	740 (35.2)	962 (45.9)
Finished secondary (10 or more classes)	189 (9.0)	211 (10.1)
HBV vaccination status of children		
Vaccine card available	352 (16.8)	953 (45.4)
Number of HBV vaccine doses received‡		
0	1,920 (91.4)	26 (1.2)
1	6 (0.3)	20 (1.0)
2	5 (0.2)	35 (1.7)
3	58 (2.8)	1,978 (94.2)
Unknown	111 (5.3)	41 (2.0)

HBV = hepatitis B virus; IQR = interquartile range.

* Born from April 1, 2001 to March 31, 2002.

† Born from November 1, 2005 to October 31, 2006.

‡ According to vaccination card or child guardian’s reporting.

Vaccination cards were available from 352 (16.8%) of the prevaccine era children and 953 (45.4%) of the vaccine era children. According to vaccination card or guardian recall, 58 (2.8%) of the prevaccine era children and 1,978 (94.2%) of the vaccine era children had received three doses of hepatitis B vaccine (Table 1). Among the vaccine era children who had a vaccination card available, hepatitis B vaccine coverage (three doses) was 96%; the vaccine coverage was 92% among who did not have vaccination card available.

### Hepatitis B virus infection prevalence.

#### Prevaccine era group.

Among the 2,100 prevaccine era children, 110 (5.2%) had evidence of HBV infection—25 (1.2%) were both anti-HBc and HBsAg positive, two (0.1%) were anti-HBc negative and HBsAg positive, and 83 (4.0%) were anti-HBc positive and HBsAg negative (Table 2). Among the 27 HBsAg-positive children, 26 had chronic HBV infection (IgM anti-HBc negative) and one had acute HBV infection (IgM anti-HBc positive). The prevalence of chronic HBV infection was 1.2% (95% CI: 0.8–1.7%). None of the prevaccine era children who were positive for anti-HBc or HBsAg had received hepatitis B vaccine. According to bivariate analysis, HBV infection prevalence was significantly higher among children who had a history of surgery (8.6%) than among children with no history of surgery (4.8%) (*P* = 0.03). The HBV infection prevalence was similar among prevaccine children with and without percutaneous exposures including receiving an injection, circumcision (males only), and ear–nose–body piercing (females only) (Table 3).

**Table 2:** Hepatitis B seroprevalence among participating children, hepatitis B seroprevalence study—Bangladesh, 2011–2012

Test	Prevaccine era children (*N* = 2,100) *n* (%)	Vaccine era children (*N* = 2,100) *n* (%)
HBV infection	110 (5.2%) (95% CI: 3.9–6.6%)	16 (0.8%) (95% CI: 0.04–1.2%)
Anti-HBc (+) and HBsAg (+)	25 (1.2%) (95% CI: 0.7–1.6%)	1 (0.05%) (95% CI: 0–0.1%)
Anti-HBc (−) and HBsAg (+)	2 (0.1%) (95% CI: 0–0.2%)	0%
Anti-HBc (+) and HBsAg (−)	83 (4.0%) (95% CI: 2.8–5.1%)	15 (0.7%) (95% CI: 0.03–1.1%)

Anti-HBc = hepatitis B core antigen; CI = confidence interval; HBsAg = hepatitis B surface antigen; HBV = hepatitis B virus. HBV infection was defined as a positive HBsAg result or a positive anti-HBc result; (+) = positive; (−) = negative.

**Table 3:** HBV infection* among prevaccine era children by selected background characteristics—Bangladesh, 2011–2012

Characteristics	Prevaccine era children (*N* = 2,100)	HBV infection (*N* = 110)	Odds ratio (95% CI)	*P* value†
Residence				
Rural	1,680	82 (4.9%)	1	
Urban	420	28 (6.7%)	1.4 (0.7–2.7)	0.32
Monthly expense > 6,000 Bangladeshi Taka				
No	1,052	53 (5.0%)	1	
Yes	989	52 (5.3%)	1.0 (0.7–1.6)	0.84
Mothers’ education				
< 5 class	1,171	72 (6.2%)	1	
5 or more classes	929	38 (4.1%)	0.6 (0.4–1.0)	0.06
History of surgery				
No	1,879	91 (4.8%)	1	
Yes	221	19 (8.6%)	1.8 (1.0–3.3)	0.03
Previously taken to a dentist				
No	1,972	104 (5.3%)	1	
Yes	128	6 (4.7%)	0.9 (0.4–2.0)	0.76
Previously received an injection (except EPI vaccination)				
No	1,131	54 (4.8%)	1	
Yes	969	56 (5.8%)	1.2 (0.8–1.8)	0.30
Circumcised (males only)				
No	337	14 (4.5%)	1	
Yes	748	37 (5.0%)	1.1 (0.5–2.3)	0.76
Ear-nose-body piercing (females only)				
No	324	15 (4.6%)	1	
Yes	691	43 (6.2%)	1.4 (0.7–2.7)	0.36

Anti-HBc = hepatitis B core antigen; CI = confidence interval; HBsAg = hepatitis B surface antigen; HBV = hepatitis B virus. Only three children had blood transfusion but did not have HBV infection and, therefore, blood transfusion was not reported as a risk factor.

* HBV infection is defined as a positive HBsAg result or a positive anti-HBc result.

† Cluster adjusted.

#### Vaccine era group.

Among the 2,100 vaccine era children, 16 (0.8%) had evidence of HBV infection—one (0.05%) child was both anti-HBc and HBsAg positive, and the remaining 15 (0.7%) children were anti-HBc positive and HBsAg negative (Table 2). The HBsAg-positive child was chronically infected (IgM anti-HBc negative) and had received three doses of hepatitis B vaccine according to the vaccination card. All 16 anti-HBc–positive children had received three doses of hepatitis B vaccine either according to the vaccination card (6/16) or guardian recall (10/16). Of the 2084 anti-HBc–negative children, 1,439 (68.5%) had detectable anti-HBs (Figure 2). Among the 1,962 children who were anti-HBc negative and had received all three doses of hepatitis B vaccine, 1,361 (69.4%) had detectable anti-HBs. Anti-HBs prevalence was 72.8% among the children who had vaccination card available.

### Hepatitis B virus infection among mothers.

Among 26 mothers of HBsAg-positive children tested (two mothers refused to provide a blood sample), four (15.4%) were HBsAg positive, and among 54 mothers of HBsAg-negative children tested, two (3.7%) were HBsAg positive (Figure 3). Although not statistically significant, mothers of HBsAg-positive children were more likely to be HBsAg positive than mothers of HBsAg-negative children (OR = 6.6; 95% CI: 0.7–60.9). Of the 21 mothers of HBsAg-positive children tested for anti-HBc, 19 (90.5%) were anti-HBc positive. Of the 47 mothers of HBsAg-negative children tested for anti-HBc, 17 (36.2%) were anti-HBc positive. Among the four HBsAg-positive mothers of HBsAg-positive children, one mother was HBeAg positive. Both of the HBsAg-positive mothers of HBsAg-negative children were HBeAg negative (Figure 3). The mother of the vaccine era child who was HBsAg positive was negative for HBsAg.

### Sensitivity and specificity of point-of-care HBsAg testing.

Of the 28 children positive by HBsAg ELISA test, 25 children were positive by point-of care HBsAg tests with whole blood tested immediately after collection of blood samples. All of the six mother samples positive by HBsAg ELISA test were positive by point-of-care HBsAg tests. Of the three samples that had discordant results between point-of-care and ELISA test (initial point-of-care test negative but ELISA positive), we retested the point-of-care HBsAg test with stored sera and found all of them to be positive. All the negative samples in HBsAg ELISA test for both mothers and children were also negative in the rapid test with whole blood. The overall sensitivity of the Determine point-of care test of HBsAg with whole blood was 91.2% (95% CI: 76.3–98.1%) and the specificity was 100% (95% CI: 99.9–100%) using the results of the ELISA test as a gold standard.

## DISCUSSION

This is the first evaluation of the seroprevalence of HBV infection after the introduction of hepatitis B vaccine in Bangladesh. The results show that the program was highly successful in reducing chronic HBV infection among children. In the prevaccine birth cohort, chronic HBV infection prevalence was 1.2%, and in the vaccine birth cohort, chronic HBV infection prevalence was 0.05%, a 96.3% (95% CI: 93.0–97.8%) reduction in HBsAg prevalence. We also found that the program achieved high vaccination coverage (94.2%) with three doses of hepatitis B vaccine, similar to previous estimates by WHO and the United Nations Children’s Fund (UNICEF).^25^

The survey results suggest that even without a birth dose, the hepatitis B vaccine program in Bangladesh was highly effective in preventing chronic HBV infection among children who received a 3-dose series. Only one child was HBsAg positive among the 2,100 vaccine era children. Among the prevaccine era HBsAg-positive children, only 15% had an HBsAg-positive mother, although it is possible that a proportion of mothers were HBgAg positive at the time of the child’s birth about 10 years earlier.^26^ Globally, a comprehensive strategy to control chronic HBV infection through vaccination is recommended even in low endemic countries, including universal vaccination of all newborn babies within 24 hours of life, considering the high rates of chronic HBV infection when HBV is transmitted at birth.^4,27^ In Bangladesh, we found a low seroprevalence of HBsAg among vaccinated children despite the lack of a birth dose. Given competing health priorities, limited resources, and the logistic challenges posed by the high rate of home births, these findings suggest that Bangladesh might not need to prioritize birth dose introduction at this time. Additional data would be useful to assess the risk of mother-to-child transmission, including the prevalence of HBsAg and HBeAg among pregnant women.

The HBsAg prevalence among prevaccine era children, aged 10–11 years (1.2%), was lower than the findings from previous small-scale studies in Bangladesh conducted before the introduction of hepatitis B vaccine. Most of the previous studies involved high-risk groups, participants in hospitals, or participants in specific urban areas. A study conducted in 1997–1998 among participants attending the outpatient department of a hospital for prevaccination HBsAg screening reported HBsAg prevalence as 5.4% among children less than 10 years^20^; another study conducted in 2005–2006 in an impoverished area of Dhaka, which is known for its high burden of infectious diseases,^18,28,29^ found an HBsAg prevalence of 12.5% among children less than 5 years.^18^ However, a much lower rate (0.8%) was observed by another study conducted in 1995 in Dhaka among school children aged 6–15 years.^30^ These findings might not be comparable with our findings because of their sample selection nature. One factor that might be related to the lower prevalence of HBsAg seropositivity among the prevaccine era children in our study is the improvement of using disposable syringes at primary health-care units and other facilities in Bangladesh in recent years. A study in Bangladesh observed 480 injection events in 24 subdistrict-level health facilities selected from all over Bangladesh; use of a new disposable syringe and needle was observed among 85% events.^31^ In addition, because of the low prevalence of HBsAg among the vaccine era cohort, there was less opportunity for the younger age children to infect the older age group.

Prevaccine era children who had previous history of surgery were more likely to have laboratory-confirmed HBV infection. This finding suggests that HBV transmission was occurring in health-care settings in Bangladesh which put patients at risk of hepatitis B and other blood-borne pathogens. No other potential exposures were significantly associated with HBV infection among prevaccine era children. We were not able to assess the risk of household-level transmission of HBV because HBsAg testing was not conducted for other household members. Additional studies will be required to better define the transmission dynamics and risk factors of HBV infection among children in Bangladesh.

We found that Determine^™^ point-of-care testing for HBsAg was comparable to standard ELISA testing (sensitivity: 91%; specificity: 100%) as has been seen in other studies.^22,32–34^ To our knowledge, this is the largest study to test the sensitivity and specificity of a point-of-care HBsAg test using whole blood in a field setting. A nationwide hepatitis B serosurvey conducted in Cambodia suggested that point-of-care HBsAg testing was feasible to use to conduct serosurveys in low-income country settings with minimal laboratory infrastructure and limited financial resources.^35^ A point-of-care HBsAg test might be useful in future serosurveys, given the expense and the logistical difficulties of collecting intravenous blood samples and maintaining the cold chain while transporting the samples back to a laboratory for ELISA testing. However, point-of-care testing with 91% sensitivity might not be adequate for clinical diagnosis of HBV infection.

We note certain study limitations. Vaccination cards were not available for a large number of children. In those cases, vaccination status of children was determined from guardians’ report that might have been subject to recall error. Our estimation of hepatitis B vaccination coverage (94.2%) was similar to WHO/UNICEF estimation in 2006.^36^ Mothers’ HBsAg and HBeAg status can change after delivery and over time,^37,38^ and therefore, the calculated OR of the possibility of mother-to-child transmission might have been different 5–10 years after the birth of the child. Vaccine-induced immunity against HBV infection (detectable anti-HBs) wanes over time; 30.6% of vaccinated children were anti-HBs negative for five years after vaccination but a number of studies have confirmed that HBV infection rarely occurs among successfully vaccinated children even when anti-HBs concentration is less than the detection level.^39–43^ Thus, vaccinated children with anti-HBs–negative status are still most likely to be protected from HBV infection. Finally, the HBsAg test result at the laboratory of CDC, Atlanta, was considered final if there was a discordant result with the laboratory at IEDCR, Dhaka. However, only 20% (835 samples) of HBsAg-negative samples at IEDCR were retested at CDC. It was unknown if there was any false-negative case at IEDCR for the remaining 80% sample. All of the negative samples at IEDCR were also negative at CDC and, therefore, it is unlikely that the remaining 80% samples were false negative at IEDCR.

This study provides important data demonstrating the success of the hepatitis B infant immunization program in Bangladesh. Regular monitoring of vaccination coverage at the national and subnational levels will be important to ensure that the gains are sustained and areas are identified where improvements will be needed. Although we found little evidence that children whose first dose of hepatitis B vaccine was delayed until week six were at increased risk of HBV infection, it remains prudent to vaccinate children in Bangladesh at the first opportunity.^44,45^ Exploring strategies to reduce HBV transmission to patients during surgery in Bangladesh might identify additional opportunities for prevention.
